# Utility of Nitrous Acid in Ulceration of the Tongue

**Published:** 1810-11

**Authors:** E. L. Knowles

**Affiliations:** Chir. Newmarket


*To the Editors of the Medical and Physical Journal.*

GENTLEMEN,

ON perusing Dr. Hall's interesting case of a tumour on the tongue, in your Journal; it occurred to me that a similar case fell under my care, when I was an assistant, which terminated in ulceration, and was successfully cured by the exhibition of nitrous nitrous acid. As near as I can recollect, the following are the particulars: Miss C— made application to me in the year 1808, to examine her tongue, which was ulcerated to a considerable extent, and very deep; her mind was much impressed, as she conceived it to be a cancerous affection. On examination, I found the edges hardened, elevated, and irregular, and the discharge was of a fœtid nature; not the least indisposition presented itself, until the mind became influenced with the idea of cancer; and as this appearance is a characteristic of that malady, I certainly was suspicious of its partakin g of that nature. As the part had ulcerated to a great extent, an operation was out of the question, especially upon such a delicate organ; I prescribed an infusion of Fol. Conii Maculati, with Sulph. Allum et Myrrh, for a gargle, to be frequently used; and directed small doses of the Submurias Hydrarg. twice a day, with an opiate at night. These were persevered in for a considerable time without any good effect, and the ulcer rapidly augmented in size, Several sorts of gargles were afterwards applied, and likewise medicines internally, without any beneficial result. At this time, Dr. Beales, of Bury St. Edmund's, had occasion to visit a patient in the neighbourhood where Miss C——— resided, and upon my detailing the particulars of the case, politely expressed a desire to see her. When he had examined the tongue, in the most minute manner, he was of opinion that it bore the characteristic of a cancerous ulcer, and was rather doubtful of success; but as he had given the Nitrous Acid in similar cases, and the effects produced were generally favourable, he particularly wished it to be tried in this; and prescribed as follows, Acid. Nitric. Dil. ℥j. Mellis, ℥ij. Aq. Font. ℔ ij. m. capiat cochlear. iij, sæpe in die; (it was directed to be sucked through a tube, to prevent the acid from corroding the teeth) an opiate was likewise given at night, and a lotion, composed of Ext. Conii. Spr. Vin. Rect. et Aquæ, was ordered to be applied to the tongue frequently. In fourteen days after the exhibition of the acid, healthy granulations were perceived to shoot out at the bottom of the ulcer, which gradually healed from this time, and in the course of three months, (although half the tongue had been in a state of ulceration) it was perfectly well. In about ten days after the tongue was healed, a vesicle appeared upon another part, and ulcerated, but it was soon counteracted by the effects of the acid, which it was now deemed advisable to administer for some time after the cure was complete. I had but little confidence in the external remedies, as similar applications were used before. Several cases of a similar appearance have occurred in my practice of late, and I always always found the Nitrous Acid, exhibited in the above form, of considerable utility.

I apprehend that this medicine has not been mentioned by any author as being serviceable in such affections; but as I have witnessed the beneficial result arising from its exhibition in numerous cases; I take particular pleasure in promulgating it through the medium of your widely circulating publication, with the motive of inducing practitioners to give it a trial in appropriate cases, and acquaint us with the result of their experiments.

I am, &c.

E. L. KNOWLES, Chir.

